# Primary peritoneal serous psammocarcinoma, rare variant: A case report

**DOI:** 10.1016/j.gore.2023.101176

**Published:** 2023-04-07

**Authors:** Srujan Kancharla, Anne Alaniz, Pulin Kothari, Stacy Norton

**Affiliations:** a 3rd Year Medical Student at Texas A&M Intercollegiate School of Engineering Medicine, United States; b Oncologic Gynecologic Surgeon at Houston Methodist Willowbrook, United States; c Pathologist at Houston Methodist Willowbrook, United States; d Gynecologic Surgeon at Houston Methodist Willowbrook, United States

**Keywords:** Ovarian cancer, Psammocarcinoma, Primary peritoneal, Insulin dependent Type 2 Diabetes Mellitus

## Abstract

Psammocarcinoma is a rare form of serous carcinoma of the ovary or peritoneum characterized by extensive psammoma body formation seen on histology. A 49-year-old obese woman, gravida 1 para 1 with poorly controlled type 2 diabetes, presented with a history of menorrhagia. She was diagnosed with both leiomyomata and simple endometrial hyperplasia without atypia in the office. Consultation with gynecological oncology and primary gynecologist resulted in a planned hysterectomy with bilateral salpingo-oophorectomy.

Laparoscopic hysterectomy and bilateral salpingo-oophorectomy were performed by primary gynecologist. Intraoperative survey of pelvis found concerning nodular lesions seen in posterior cul-de-sac. Lesions were excised and sent for frozen section. Pathology showed invasive peritoneal epithelial implant with psammomatous calcifications. Gynecological oncologist stand-by was called in and proceeded with surgical debulking and staging procedure.

Post operatively, the patient has been diagnosed with primary peritoneal low grade serous psammomacarcinoma stage III A2. Her case has been presented to tumor board for multidisciplinary management and is now undergoing adjuvant hormonal therapy utilizing letrozole with chest, abdomen, and pelvic CT scans every 6 months. Standardized protocols are hindered by the rarity of this tumor. Benefits for this oncologic diagnosis are not clearly understood due to the rarity of this tumor.

The significance of this case presentation is to highlight the multidisciplinary approach to the workup, diagnosis, treatment (both surgical and medical) and follow up of a rare gynecologic oncologic case. While the surgical team was expecting to find at most significant pathology, an endometrial carcinoma, a rarer primary peritoneal carcinoma was found. Due to the pre surgical planning, and intraoperative teamwork of the pathology, gynecology and oncology teams, this patient received the individualized and disease specific needed surgical and medical care warranted by her unique diagnosis.

## Highlights

• With unremarkable postoperative course, patient is on letrozole therapy with routine 6 month CT scans for monitoring.
• The most agreed recommendation for treating this cancer from authors was aggressive cytoreductive surgery.
• Patient’s poorly controlled diabetes was a risk factor for developing ovarian cancer.
• This cancer has a better prognosis than invasive serous adenocarcinoma and a similar indolence as borderline ovarian tumors.
• The aggressive clinical behavior in this malignancy with distant metastasis and recurrence is rare as seen in this patient.

## 1 Introduction

Since its discovery in 1917, there have only been 31 cases reported in literature of serous psammocarcinoma of primary peritoneum (Helal et al., 2022). Serous psammocarcinoma is a rare variant of ovarian cancer characterized by massive psammoma bodies and moderate cytological atypia (Jena et al., 2015). This cancer may originate from the ovarian stroma or from primary peritoneum. Diagnosis of this carcinoma is based on histological evaluation. The evaluation is performed following removal of ovaries and/or biopsies of the peritoneum. While psammoma bodies have been found in less aggressive clinical course seen in papillary thyroid carcinoma and cranial meningioma, its prognostic significance is unclear (Alanbay et al., 2009). Patients are typically treated with surgical debulking and hormonal or chemotherapy; however, due to its low incidence, there are no standard treatment guidelines.

## 2 Case report

A 49-year-old G1P1 female with history significant for Hypertension, BMI of 31.1 and type 2 diabetes mellitus poorly controlled with Jardiance and Trulicity (HbA1c of 7.6) presented with three months of heavy menstrual bleeding that had been unresponsive to medical management in February 2022. A pelvic ultrasound showed an enlarged fibroid uterus, measuring at 10.5 × 6.3 × 6.8 cm. Endometrial biopsy was performed and showed hyperplasia without atypia and features suggestive of benign endometrial polyp. Plan of care was made for definitive surgical management by a minimally invasive hysterectomy and bilateral salpingo-oophorectomy (BSO). Intraoperatively, there was observed, on the posterior cul-de-sac and anterior of the sigmoid colon, numerous excrescences, suggestive of metastatic carcinoma. Shortly after, gynecologic oncologist was consulted and proceeded with cytoreductive surgery, which included washing and debulking. This was performed to remove as much tumor as possible and provide extensive tissue samples for our pathologist to analyze. From pathology report, the portions of interest were predominantly surface involvement of bilateral fallopian tubes and bilateral ovaries showing low grade serous psammocarcinoma. Additionally, the sigmoid colon serosa nodule, omentum, and right and left lower paracolic gutter peritoneum nodules showed low grade serous psammocarcinoma. Right and left upper paracolic gutter peritoneum nodule exhibited fibroadipose tissue with rare psammoma bodies but negative for tumor cells. The cervix, endometrium, myometrium, right and left pelvic lymph nodes did not show significance of any tumor.

Patient was diagnosed with Serous Psammocarcinoma of Primary Peritoneum, Stage III A2. Her case was presented at Multidisciplinary Gynecologic Oncology Tumor Board Conference to determine best course of treatment. From this, patient began hormonal therapy with letrozole with low dose Chest, Abdomen and Pelvic CT scans with contrast every 6 months to monitor any progression of the ovarian cancer.

**Fig. 1:**
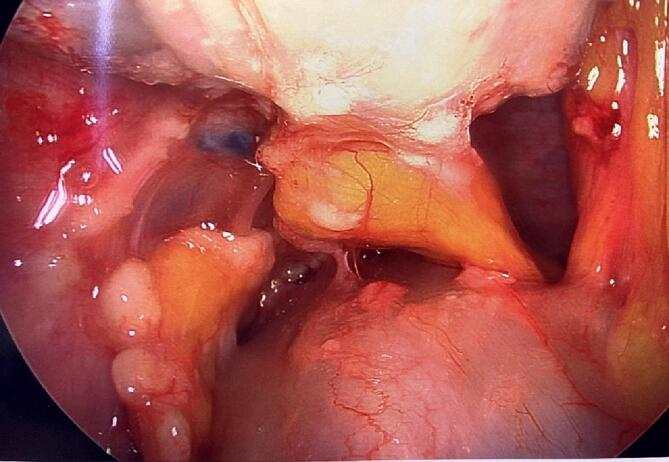
Nodular Excresences in the Posterior cul-de-sac.

**Fig. 2:**
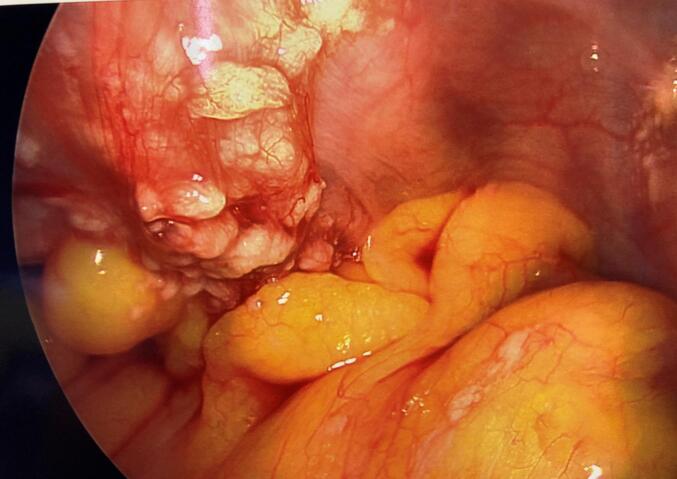
Nodular Excresences distributed along Pelvic Wall.

**Fig. 3:**
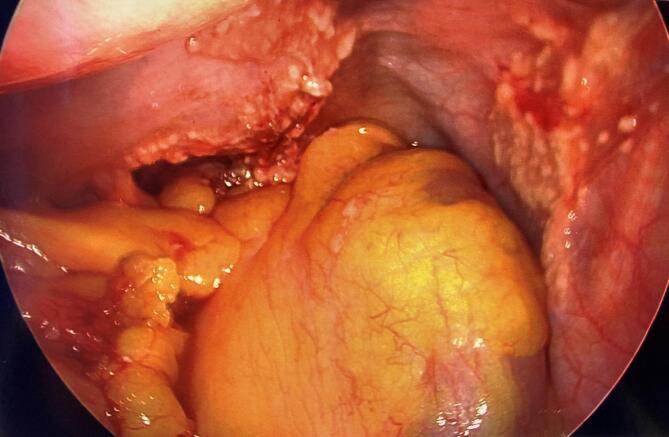
Additional protuberances seen along serosa of colon.

**Fig. 4:**
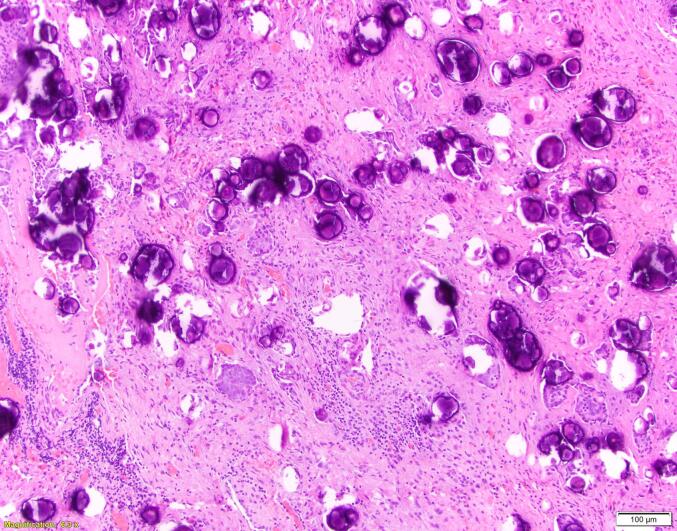
Microscopic examination (Hematoxylin and eosin staining × 40) of Psammoma body formation.

**Fig. 5:**
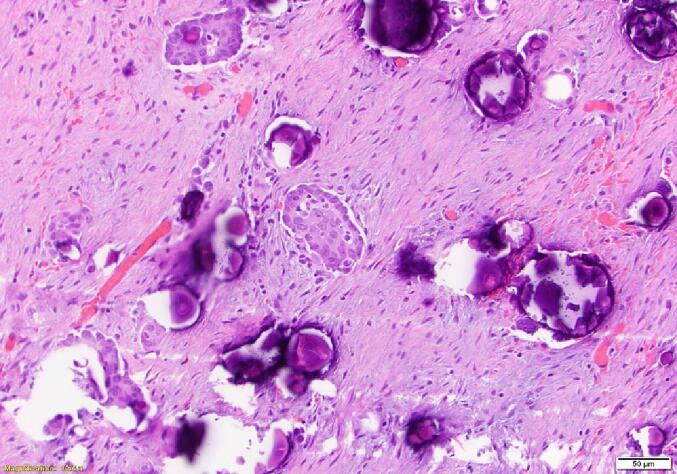
Closer Microscopic View (x?) of Psammoma body formation.

**Fig. 6:**
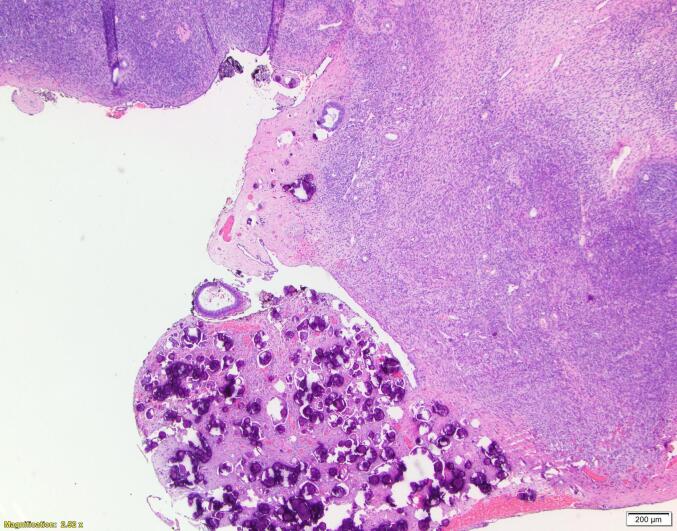
Microscopic examination (Hematoxylin and eosin staining × 40) of Psammocarcinoma - left ovary.

Post operatively, patient was in minimal abdominal pain and denied nausea, vomiting, abdominal bloating, early satiety and had no significant changes in bowel and bladder habits. Genetic testing was unremarkable with no mutation for any hereditary cancer syndromes. Nine months postoperative, there has been no evidence of metastatic disease present on CT scans and patient is asymptomatic of ovarian cancer. She experienced post-menopausal symptoms as expected side effect from letrozole with symptomatic management outpatient. CA-125 from three weeks postop down trended from 21.5 to 9.7 seven months postop (see Fig. 1, Fig. 2, Fig. 3, Fig. 4, Fig. 5, Fig. 6).

## 3 Discussion

Ovarian carcinomas pose a clinical challenge in gynecologic oncology. Ovarian cancer is the second most common gynecological cancer in the US. Patients can be asymptomatic for prolonged periods until the disease progresses to an advanced stage. In fact, more than two-thirds of these are diagnosed at an advanced stage (Motohara et al., 2010). This patient presented with symptoms of menorrhagia for 3 months refractory to medication. Fibroids and endometrial hyperplasia were diagnosed and explained her symptoms, consequently reducing suspicion of any malignancy. Post operatively, the patient had genetic testing, and no mutations were found to suggest risk of hereditary cancer.

To categorize this cancer, in 1990, Gilks et al. defined four specific histologic diagnostic criteria for psammocarcinoma: 1|) destructive invasion of the ovarian stroma or of the intraperitoneal viscera and peritoneum; 2) no more than moderate cytology atypia; 3) no areas of solid epithelial proliferation except for occasional nests, with no more than 15 cells in diameter, and 4) at least 75% of the papillae associated with or completely replaced by psammoma body formation (Gilks et al., 1990). In 1994; Chen et al. expanded Gilk’s criteria for diagnosis of peritoneal psammocarcinoma to include either invasions of the intraperitoneal viscera or an invasive pattern in the peritoneum (Chen, 1994).

From Multidisciplinary Gynecologic Oncology Tumor Board Conference, no chemotherapy was chosen for this patient and instead the patient is treated with hormonal therapy letrozole. In the review of 5 patients with primary psammocarcinoma of the ovary or peritoneum by Poujade et al., benefits of adjuvant chemotherapy were poorly defined in these low-grade tumors with a low mitotic index (Poujade et al., 2009). However, for treatment, most authors do agree and recommend aggressive cytoreduction initially, as is seen with this patient (Jena et al., 2015). The effectiveness of letrozole as hormonal maintenance therapy for ovarian cancer was evaluated by Gershenson et al. This showed that after primary treatment of cytoreductive surgery and platinum-based chemotherapy, the use of letrozole (n = 38) had significantly longer median progression-free survival than patient who underwent routine observation (n = 133) (64.9 months vs 26.4 months, p < 0.001). This benefit was independent of residual disease at completion of primary chemotherapy (Marchetti et al., 2020). Thus, to avoid harmful side effects of chemotherapy and with patient’s uncomplicated postoperative course, maintenance hormonal therapy with letrozole was chosen. As mentioned before, the CT scan of chest, abdomen and pelvis is performed every 6 months to routinely monitor for progression of the ovarian cancer. Nine months postoperative, patient has been asymptomatic with down trending CA-125 and CT scans unremarkable for signs of progression of ovarian malignancy.

Specific to this case, the patient had poorly controlled diabetes mellitus with HbA1c of 7.6. In a comprehensive meta-analysis regarding type 2 diabetes mellitus and risk of developing cancer, among 7,651 individuals, presence of diabetes is associated with a 10% increase of relative risk to develop ovarian cancer (Suh and Kim, 2019). The hyperglycemia is responsible for oxidative stress and DNA damage, and this may act as an inciting factor for tumorigenesis. Overall, post operative treatment through maintenance therapy with letrozole and routine 6-month CT scans is not standard, but this avoids the harmful side effects with chemotherapy while patient continues in a disease-free state. Should the patient experience recurrence of malignancy or onset of worsening symptoms, more aggressive treatment options including chemotherapy will be considered.

## 4 Conclusion

The difficulty in treating ovarian cancers poses as a significant challenge due to its discovery typically at an advanced stage. Though the presence of psammoma bodies is noted and have generally shown to have good prognostic value, the rarity of this incidental finding obscures this value. Based on literature review, these tumors have a better prognosis than invasive serous adenocarcinoma (Jena et al., 2015). Though most seem to follow an indolent course similar to borderline tumors of the ovary, aggressive clinical behavior with distant metastasis and recurrence is rare. However, despite the similarities, the intrinsic behavior of this disease remains unresolved. This creates challenges with the lack of protocol to treatment, but revisions to treatment is expected. Our case is treated through conservative treatment of hormonal maintenance therapy and routine CT scans. There is no recurrence of disease clinically or on radiology. Through a multidisciplinary approach, the patient will be frequently monitored through telemedicine and outpatient visits with both gynecologist, oncologist, and their expert teams to foster proactive, individualized, high quality care.

## Author contributions

Dr. Norton, gynecological surgeon, performed the surgery with Dr. Alaniz, gynecological oncologist, on assist for biopsies, wash and debulking. Dr. Norton provided figures from surgery with Dr. Kothari providing figures from pathology reports including staging and classifications of specimens. Mr. Kancharla provided 3rd assist to the surgery and wrote the manuscript. Dr. Norton, Dr. Alaniz and Dr. Kothari edited the draft. Dr. Norton approved the manuscript. All authors read, revised, and approved the manuscript.

## Declaration of Competing Interest

The authors declare that they have no known competing financial interests or personal relationships that could have appeared to influence the work reported in this paper.

## References

[b0005] Alanbay I., Dede M., Ustün Y., Karaşahin E., Deveci S., Günhan O., Yenen M.C. (2009). Serous psammocarcinoma of the ovary and peritoneum: two case reports and review of the literature. Arch. Gynecol. Obstetrics.

[b0010] Chen K.T. (1994). Psammocarcinoma of the peritoneum. Diagn. Cytopathol..

[b0015] Gilks C.B., Bell D.A., Scully R.E. (1990). Serous psammocarcinoma of the ovary and peritoneum. Int. J. Gynecological Pathol.: Off. J. Int. Soc. Gynecol. Pathol..

[b0020] Helal I., Khanchel F., Jouini R. (2022). Case Report: Fortuitous discovery of primary peritoneal psammocarcinoma. F1000 Research.

[b0025] Jena S.K., Mishra P., Mohapatra V., Singh S. (2015). Bilateral Serous Psammocarcinoma of Ovary: Rare Variant Low Grade Serous Carcinoma. Case Rep. Obstetrics Gynecol..

[b0030] Marchetti C., De Felice F., Ergasti R., Scambia G., Fagotti A. (2020). Letrozole in the management of advanced ovarian cancer: An old drug as a new targeted therapy. Int. J. Gynecol. Cancer.

[b0035] Motohara T., Tashiro H., Miyahara Y., Sakaguchi I., Ohtake H., Katabuchi H. (2010). Long-term oncological outcomes of ovarian serous carcinomas with psammoma bodies: a novel insight into the molecular pathogenesis of ovarian epithelial carcinoma. Cancer Sci..

[b0040] Poujade O., Uzan C., Gouy S., Pautier P., Duvillard P., Morice P. (2009). Primary psammocarcinoma of the ovary or peritoneum. Int. J. Gynecol. Cancer: Off. J. Int. Gynecol. Cancer Soc..

[b0045] Suh S., Kim K.W. (2019). Diabetes and Cancer: Cancer Should Be Screened in Routine Diabetes Assessment. Diabetes Metab. J..

